# The efficacy and safety of vagus nerve stimulation in pediatric epilepsy in China: a multicenter cohort study

**DOI:** 10.1186/s42494-025-00221-7

**Published:** 2025-12-02

**Authors:** Wenyu Liu, Wenjing Li, Chenyang Zhao, Xintong Wu, Dong Zhou

**Affiliations:** Departments of Neurology, West China Hospital, Sichuan University, Chengdu, 610041 People’s Republic of China

**Keywords:** Efficacy, Epilepsy, VNS, Children

## Abstract

**Background:**

The field of epilepsy neural regulation represented by VNS is rapidly developing. Our aim was to investigate the safety and effectiveness of vagus nerve stimulation (VNS) as an adjunct therapy for pediatric epilepsy in a multi-center study across China.

**Methods:**

Children with epilepsy undergoing VNS as supplementary treatment were consecutively enrolled in this study. Eligibility was limited to children aged 1–16 years with a confirmed epilepsy diagnosis, a stable antiseizure medication regimen, and a minimum of two seizures per 28-day cycle during the 8-week retrospective baseline.

**Results:**

Eighty-seven children (54 males; mean age 8.21 ± 3.88 years, range 0–16) were included, with seizures beginning at an average age of 3.03 ± 2.90 years. A ≥ 50% reduction in seizure frequency was observed in 23.7% at 6 weeks, 20.3% at 10 weeks, 22.6% at 18 weeks, and 18.6% at 26 weeks. Seizure freedom was achieved in 17.1%, 15.9%, 11.3%, and 16.3% of patients at the same intervals. Two subjects experienced adverse events, both of which were mild and transient.

**Conclusions:**

VNS demonstrated moderate efficacy and a favorable safety profile as an adjunct treatment in children with epilepsy. Further large-scale, long-term studies are recommended to confirm these findings.

## Background

Epilepsy affects 0.5–1% of children, with early childhood being the most frequent period of onset [1, 2]. Although anti-seizure medication (ASM) is the primary treatment, many patients continue to experience seizures [3]. Drug resistance occurs in about one-third of cases, especially in focal epilepsy [4], and treatment becomes even more challenging in severe pediatric epilepsy syndromes [5].

In 1994, vagus nerve stimulation (VNS) was approved in Europe for the treatment of refractory epilepsy with focal or generalized seizures. In 1995, a randomized double-blind controlled trial (RCT) of vagus nerve surgery for the treatment of refractory epilepsy was published in the journal Neurology [6], which led to the approval of VNS as an adjuvant therapy for drug-resistant focal epilepsy in adolescents over 12 years old and adults by the US FDA (Food and Drug Administration) in 1997. So far, vagus nerve stimulation has been used in over 75 countries and more than 130,000 patients worldwide. VNS surgery has been widely recognized in the field of epilepsy surgery for its advantages of minimal trauma and wide applicability, and has become a commonly used surgical method in epilepsy surgery abroad. And as a result, many other neural regulation techniques have been developed, including reactive neural stimulation (RNS) of the epileptic area and deep brain stimulation of the anterior thalamic nucleus (ANT-DBS) [7, 8]. More recently, closed-loop VNS systems have been developed to deliver stimulation automatically, triggered by seizure-related changes in heart rate such as ictal tachycardia [9, 10]. Therefore, the field of epilepsy neural regulation represented by VNS is rapidly developing.

The indications of VNS in epilepsy are also constantly expanding. In 2017, the US FDA further approved the use of VNS therapy system as an adjuvant therapy for children aged 4 and above with partial epileptic seizures that are difficult to cure with ASMs. At present, age is no longer a limiting condition for the use of VNS. Fernandez [10] evaluated VNS therapy in 17 children under the age of three with refractory epilepsy. After one year of treatment, 33% (5 of 15) showed significant seizure reduction. Importantly, the therapy was well tolerated across all cases, with no serious side effects reported.

VNS involves the delivery of controlled electrical pulses to the vagus nerve via an implanted device. This stimulation modulates brain activity indirectly and has been shown to reduce seizure frequency and intensity. Since being approved by the FDA in 1997, VNS has become an important adjuvant therapy for drug-resistant epilepsy. The indications of VNS includes: 1. Drug-resistant epilepsy: suitable for patients who have not been treated with at least two ASMs. 2. Not suitable for patients undergoing resection surgery, such as those with epilepsy lesions located in functional areas, multifocal, or those whose lesions cannot be accurately located. 3. Special epilepsy syndrome: Lennox-Gastaut syndrome, nodular sclerosis related epilepsy, etc. 4. Child patients: especially suitable for children who cannot undergo brain surgery (FDA approved age ≥ 4 years old).

The mechanism of action includes: firstly, neurotransmitter regulation: stimulating the vagus nerve can increase the release of norepinephrine, serotonin, and GABA, and inhibit abnormal discharges. Secondly, brain network regulation: it affects the solitary tract nucleus, thalamus, and limbic system, reducing synchronous abnormal electrical activity. Thirdly, anti-inflammatory effects: VNS may alleviate epilepsy related neuroinflammation by inhibiting inflammatory factors. This study aimed to evaluate the efficacy and tolerability of VNS as a treatment for pediatric epilepsy patients.

## Methods

### Ethical approval

This study was conducted as a multicenter collaboration across 86 epilepsy centers distributed throughout mainland China. The participating centers included tertiary hospitals and specialized pediatric neurology departments located in major regions such as Beijing, Shanghai, Guangdong, Sichuan, Hunan, Shandong, Jiangsu, and Zhejiang, among others. These centers represent a broad geographic range and diverse patient populations, encompassing both urban and provincial areas. Most of the centers are affiliated with academic medical institutions or regional children's hospitals with established experience in the diagnosis and treatment of pediatric epilepsy. Ethical clearance was secured from review boards at 86 epilepsy centers across China. Informed consent was obtained from legal guardians, and assent was additionally required from children aged 4 years and older. For minors, both the child and the guardian signed the consent documentation. Of 141 children screened, 87 were included after excluding 54 due to incomplete follow-up.

### Participants

Eligibility was limited to children aged 1–16 years with a confirmed epilepsy diagnosis, a stable ASM regimen, and a minimum of two seizures per 28-day cycle during the 8-week retrospective baseline. Exclusion criteria included psychogenic non-epileptic events, psychiatric disorders, substance abuse, use of interfering medications, ketogenic dietary therapy, prior or scheduled brain surgery within four months, or lack of consent.

### Study design and outcomes

Baseline assessments and follow-ups at 6, 10, 18, and 26 weeks post-VNS were conducted. Medication regimens remained unchanged from four weeks prior to enrollment through study completion. All drugs and dosages were recorded at each visit. The use of sedatives, antidepressants, and Chinese patent medicines for epilepsy was prohibited. Primary outcomes were defined as changes in seizure frequency and the proportion of patients achieving a ≥ 50% seizure reduction during the 4-week maintenance phase compared to baseline. Secondary endpoints included seizure-free rates and treatment retention. Adverse events were graded using standard criteria, and severe events prompting withdrawal were documented.

### Statistical analysis

Continuous variables (e.g., age, age at seizure onset, physical metrics, seizure counts, and drug dosage) were assessed for normality using the Shapiro–Wilk test. Variables that followed a normal distribution were expressed as means ± standard deviations and compared using *t*-tests. Non-normally distributed continuous variables were summarized as medians (interquartile range) and analyzed using the Wilcoxon signed-rank test. Categorical variables were expressed as counts and percentages, and comparisons were performed using the chi-square test or Fisher’s exact test where appropriate (i.e., when expected cell counts were < 5). All statistical analyses were conducted using SPSS version 26.0 (IBM Corp., Armonk, NY), with a two-tailed *P* value of < 0.05 considered statistically significant.

## Results

### Demographic characteristics

Of the 87 children included, 54 (62.1%) were male. The mean age at enrollment was 8.21 ± 3.88 years, with seizures beginning at a mean age of 3.03 ± 2.90 years. A family history of epilepsy was identified in 3 patients, and comorbidities were present in 73 cases (Table 1).

**Table 1:** Clinical characteristics of subjects

Characteristics	*N* = 87
Age (years)	8.21 ± 3.88
Sex (male/female), *n* (%)	
Male	59 (67.82)
Female	28 (32.18)
Age at onset (years)	3.03 ± 2.90
Family history (±), *n* (%)	
+	3 (3.45)
-	84 (96.55)
Seizure type, *n* (%)	
Focal-only	34 (39.08)
FBTCS	8 (9.20)
Comorbidities	73 (83.9)

Values are shown as mean ± standard deviation (SD) or *n*

*Abbreviations*: *FBTCS* focal to bilateral tonic–clonic seizures

### Primary outcomes

#### Seizure frequency

##### ≥50% response rate

The proportion of patients with a ≥50% reduction in seizure frequency, relative to baseline, was 23.7% at visit 2, 20.3% at visit 3, 22.6% at visit 4, and 18.6% at visit 5 (Table 2).

**Table 2:** Seizure frequency change rate for visits 2–5 compared to visit 1

	Visit 1	Visit 2	Visit 3	Visit 4	Visit 5
Seizure free, *n* (%)	1 (1.1)	3 (3.9)	3 (4.3)	3 (5.7)	4 (9.3)
75% reduction, *n* (%)	8 (9.2)	12 (15.8)	13 (18.8)	16 (30.2)	12 (27.9)
50% reduction, *n* (%)	16 (18.4)	18 (23.7)	14 (20.3)	12 (22.6)	8 (18.6)
25% reduction, *n* (%)	15 (17.2)	16 (21.1)	19 (27.5)	14 (26.4)	9 (20.9)
No change, *n* (%)	45 (51.7)	25 (32.9)	18 (26.1)	8 (15.1)	10 (23.3)
Increase, *n* (%)	2 (2.3)	2 (2.6)	2 (2.9)	0 (0.0)	0 (0.0)
χ^2^	-	104	69.2	26.8	44.2
*P*	-	< 0.001	< 0.001	0.140	0.001

### Secondary outcome measures

#### Seizure-free rate

From visit 2 to visit 5, seizure-free rates compared to visit 1 were 17.1%, 15.9%, 11.3%, and 16.3%, respectively (Table 3).

**Table 3:** Seizure-free rate for visits 2–5 compared to visit 1

	Seizure (%)	Seizure free (%)	χ^2^	*P*
Visit 1	75 (84.3)	14 (15.7)	-	-
Visit 2	63 (82.9)	13 (17.1)	0.057	0.836
Visit 3	58 (84.1)	11 (15.9)	0.001	0.971
Visit 4	47 (88.7)	6 (11.3)	0.534	0.619
Visit 5	36 (83.7)	7 (16.3)	0.007	0.936

#### Adverse events

Mild dizziness was reported in two patients. No severe adverse events were recorded.

## Discussion

This study is the first multi-center cohort in China to evaluate VNS in children with refractory epilepsy. All participants had previously failed standard pharmacological therapy. Findings demonstrate that VNS is both an effective and well-tolerated adjunctive treatment in this population.

Epilepsy is a chronic and diverse disorder, frequently beginning in childhood and characterized by recurrent seizures. Although anti-seizure medications are the mainstay of treatment, they often fail to achieve lasting control, require long-term use, and may cause adverse effects. While some children respond to alternatives such as surgery, neuromodulation, or ketogenic diets, comorbidities remain common and often impair cognitive function and quality of life.

VNS has been applied in clinical practice for 30 years, and a large number of literature and studies have shown its definite efficacy, which can reduce the frequency and severity of epileptic seizures, and is both effective and safe. The American Academy of Neurology (AAN) guidelines released in 2013 [11] confirmed the efficacy of VNS through a summary analysis of 14 Class III studies, with approximately 55% to 65% of patients experiencing a 50% reduction in seizures and approximately 6% to 11% achieving complete control of seizures [11, 12]. A meta-analysis based on 1061 patients from 16 clinical studies in 2019 suggested that 53% of patients experienced a reduction of over 50% in seizures [13]. A systematic review of VNS treatment for children with refractory epilepsy, which included 101 studies in 2021, showed that 56.4% of patients experienced a 50% or more reduction in seizures, 11.6% achieved seizure free outcomes, and fewer attempts of antiepileptic drugs before VNS were associated with fewer seizures after VNS surgery, suggesting the possibility of early addition of VNS for better therapeutic effects [14]. A retrospective cohort study of 347 pediatric and adolescent patients showed that the effective rates for children under 12 years old at one and two years were 43% and 50%, respectively, and the seizure free rates were 7.8% and 11.3%, respectively [15]. In addition to reducing seizure frequency, VNS may also alleviate the severity of epileptic seizures and shorten their duration [11, 15]. And the anti-epileptic efficacy of VNS has a time cumulative effect.

There are currently reports on the efficacy of certain specific epilepsy syndromes, epileptic encephalopathy, and epilepsy caused by specific etiologies. The effective rate of Lennox-Gastaut syndrome is 25% to 78% [16], and a meta-analysis of 68 Dravet syndrome patients showed that 52.9% of patients had a 50% reduction in epileptic seizures [17]. Another meta-analysis on VNS treatment for epilepsy: VNS was used for generalized seizures, resulting in a 57.5% reduction in seizure frequency; focal attacks decreased by 42.5%; other types of seizures decreased by 53.7%; VNS is used for patients with post-traumatic epilepsy, reducing seizure frequency by 78.6%; patients with tuberous sclerosis epilepsy have a 68.1% reduction in seizure frequency [18]. There are also small sample reports on the treatment of hereditary epilepsy such as Rett syndrome, Doose syndrome, and idiopathic generalized epilepsy [19]. There are also reports of using VNS therapy for the treatment of refractory status epilepticus [20].

The therapeutic effect and other benefits of vagus nerve stimulation on comorbidities of epilepsy. Treating epilepsy aims to control its occurrence, but the treatment of epilepsy comorbidities is equally important. Depression is common among epilepsy patients, and research suggests that VNS can improve depression scores by 25% to 35%, anxiety scores by 35%, and emotional scores by 25%. Therefore, for epilepsy patients with comorbid depression, VNS therapy can be recommended [21, 22]. A cohort study found [23] that VNS treatment can reduce the incidence of sudden epileptic death (SUDEP). Patients treated with VNS had a SUDEP rate that decreased from 5.5 ‰ to 1.7 ‰ 2 years later. In addition, a large sample study [24] conducted a survey on the quality of life of patients through a doctor's questionnaire, and showed that 58% to 63% of patients with VNS implantation had improved alertness, 43% to 49% had improved emotions, 38% to 45% had improved language communication, 29% to 39% had improved school and career achievements, and 29% to 39% had improved memory after more than one year. Other studies have also reached similar conclusions [25–27].

Domestic research status and development trend of vagus nerve stimulation surgery. In recent years, research on domestic VNS has gradually increased. In 2014, China completed the registration clinical trials of domestic VNS [30], and in 2016, domestic VNS began to be applied in clinical practice. Currently, over 140 hospitals in China have implemented VNS therapy, and nearly 5000 epilepsy patients have received this treatment. In 2010, Meng et al [28] reported 21 cases of VNS-treated refractory epilepsy, followed up for 4–16 months, and 10 cases showed a reduction of over 50% in seizures. In 2015, Liu et al [29] reported 71 cases and followed up for 3–56 months. Among them, 51 cases (71.83%) had a reduction of more than 50% in epileptic seizures, and 14 cases had complete seizure cessation. Chinese scholars Zhao Meng et al [28] recently performed VNS treatment on 43 children with post encephalitis epilepsy and completed a 43-month follow-up. The postoperative language, interpersonal communication, learning/work ability, and memory of the patient have significantly improved compared to preoperative levels.

China began to apply VNS to patients with drug-resistant epilepsy in the 1990s. In 2000, China officially approved VNS for the treatment of drug-resistant epilepsy, especially for patients with focal seizures, which can effectively decrease the seizure frequency and intensity. However, compared with the widespread application of VNS abroad, VNS has only been used in thousands of surgeries in China, and the imported vagus nerve stimulator used is expensive, which is one of the main reasons limiting the clinical application of vagus nerve stimulators; VNS is ineffective for a very small number of patients, and its mechanism of action still needs further clarification. More importantly, there is still a lack of standard evaluation procedures for vagus nerve stimulator usage in epilepsy treatment in China. A series of standard operating procedures for disease diagnosis, surgery, program control, and patient management have not yet been popularized, and a complete system for personnel training, equipment maintenance, and center evaluation is also extremely lacking. Therefore, there is an urgent need to establish standardized preoperative evaluation, optimal indications, postoperative programmable plans, and guiding principles for vagus nerve electrical stimulation therapy for epilepsy. Furthermore, a large-scale clinical trial of vagus nerve stimulation for the treatment of epilepsy will be conducted, with the promotion, validation, evaluation, and rational improvement of clinical application standards.

At present, the efficacy of VNS treatment for refractory epilepsy in children and adults is confirmed both domestically and internationally. For refractory epilepsy patients who cannot undergo surgical resection, it is an effective choice, and its treatment effect can be maintained for a long time. With the accumulation of clinical research, there are additional therapeutic effects on comorbidities with epilepsy such as mood disorders, depressive episodes, sudden death from epilepsy, and improvement of quality of life. However, VNS has not yet been widely applied in China. Vagus nerve stimulation therapy for epilepsy usually lasts for several years. Only standardized preoperative evaluation, optimal indications, postoperative programmable plans, and guiding principles for epilepsy vagus nerve electrical stimulation therapy can be formed; further promote validation, efficacy evaluation, and rationalization improvement. This requires the establishment of a series of standard operating procedures for disease diagnosis, surgery, program control, patient management, as well as a complete system for personnel training, equipment maintenance, and center evaluation. This project will construct a network database and remote follow-up program control system, which will be integrated into hospitals at different levels to form a hierarchical diagnosis and treatment system, and jointly achieve the widespread and reasonable application of vagus nerve stimulation.

Limitations of this study include a brief follow-up period, small sample size, and incomplete cognitive data. To confirm the sustained benefits of VNS, long-term, large-scale prospective trials are needed. Future research should also explore its impact on cognitive outcomes, emotional health, and overall well-being. Notably, this is the first study to report on VNS using a domestically produced device in Chinese pediatric patients, reflecting its increasing clinical adoption.

## Conclusions

Advancements in broad-spectrum antiseizure medications and neuromodulation have expanded treatment options for epilepsy. VNS has shown particular promise as an adjunct therapy for drug-resistant cases. This multicenter prospective study found that a domestically produced generic VNS device was effective and well-tolerated in Chinese children with refractory epilepsy. Further large-scale and long-term studies are required to confirm these outcomes.

## Data Availability

All data are available from the corresponding author upon reasonable request.

## Abbreviations

AAN: The American Academy of Neurology
ANT-DBS: Deep brain stimulation of the anterior thalamic nucleus
ASM: Anti-seizure medication
FDA: Food and Drug Administration
RCT: Randomized controlled trial
RNS: Reactive neural stimulation
VNS: Vagus nerve stimulation
